# The role of financial factors in the mobility and location choices of General Practitioners in Australia

**DOI:** 10.1186/s12960-019-0374-4

**Published:** 2019-05-24

**Authors:** Michelle McIsaac, Anthony Scott, Guyonne Kalb

**Affiliations:** 1 World Health Organization, Avenue Appia 20, 1293 Geneva, Switzerland; 2 Melbourne Institute of Applied Economic and Social Research, The University of Melbourne, 111 Barry Street, Carlton, VIC 3053 Australia

**Keywords:** Geographic mobility, Financial incentives, Labour market

## Abstract

**Background:**

The geographic distribution of health workers is a pervasive policy concern. Many governments are responding by introducing financial incentives to attract health care workers to locate in areas that are underserved. However, clear evidence of the effectiveness of such financial incentives is lacking.

**Methods:**

This paper examines General Practitioners’ (GPs) relocation choices in Australia and proposes a dynamic location choice model accounting for both source and destination factors associated with a choice to relocate, thereby accounting for push and pull factors associated with job separation. The model is used to simulate financial incentive policies and assess potential for such policies to redistribute GPs. This paper examines the role of financial factors in relocating established GPs into neighbourhoods with relatively low socioeconomic status. The paper uses a discrete choice model and panel data on GPs’ actual changes in location from one year to the next.

**Results:**

This paper finds that established GPs are not very mobile, even when a financial incentive is offered. Policy simulation predicts that 93.2% of GPs would remain at their current practice and that an additional 0.8% would be retained or would relocate in a low-socioeconomic status (SES) neighbourhood in response to a hypothetical financial incentive of a 10% increase in the earnings of all metropolitan GPs practising in low-SES neighbourhoods.

**Conclusion:**

With current evidence on the effectiveness of redistribution programmes limited to newly entering GPs, the policy simulations in this paper provide an insight into the potential effectiveness of financial incentives as a redistribution policy targeting the entire GP population. Overall, the results suggest that financial considerations are part of many factors influencing the location choice of GPs. For instance, GP practice ownership played almost as important a role in mobility as earnings.

## Background

Policymakers have been concerned with the supply and distribution of health workers for decades [26, 28]. In response, governments all over the world have introduced a range of policies to encourage primary health care workers to locate in areas that are underserved [10, 28].

Financial incentives to influence the recruitment and retention of health workers to underserved areas are becoming a widespread policy option; however, clear evidence of their effectiveness is lacking [5, 13, 35]. The studies that are available rely on cross-sectional data of newly entering doctors; they suggest that financial incentives should effectively distribute newly trained doctors into underserved areas [3, 15]. However, the retention of doctors, particularly General Practitioners (GPs), in underserved areas remains an unresolved issue [27]. Therefore, there is a need to examine the impact of financial incentives on the mobility of all GPs, not just newly entering GPs.

Discrete choice experiments (DCEs) have demonstrated that a range of non-pecuniary factors such as hours worked, on-call hours, and patient mix play an important role in GPs’ location choices [30]. These non-pecuniary factors have, thus far, been overlooked in policy evaluations of financial incentives aiming to induce GP relocation.

This study is set in Australia where GPs are paid by fee-for-service and can charge patients what the market will bear. Patients can claim a fixed subsidy from Medicare, the tax-financed national medical insurer. The difference between the fee charged and the subsidy is the out-of-pocket cost born by the patient; it is not covered by private health insurance. Around 80% of GP visits are charged at the level of the Medicare subsidy (i.e., bulk-billed) [8]. Australia, like many countries, is facing a continuing problem with equity of access to health services by vulnerable patient groups of low-socioeconomic status (SES) [11, 25]. Likely representing an example of the inverse care law, where medical care is least likely to reach those most in need [14].

This paper examines the role of financial factors in relocating established GPs into metropolitan neighbourhoods with low-socioeconomic status. GPs’ decisions to locate largely in affluent areas can result in inefficiencies in the allocation of health resources [24]. The paper uses a discrete choice model and panel data on GPs’ actual observed changes in location from one year to the next. The model accounts for several non-pecuniary practice attributes and a range of personal characteristics. Incorporating the dynamic aspects of location choice leads to a more accurate and relevant assessment of the importance of financial factors than what is currently available. Once all these aspects are accounted for, a policy simulation suggests that financial incentives are not very effective at inducing established GPs to relocate.

## Methods

There are around 25 000 GPs in Australia. In 2008, approximately 36% of GPs practised as principals and 12% as associates, 7% were salaried, and 40% were contractual employees, while 2% were practising as locums and 3% were working in other areas such as at universities.^1^ Principal GPs (co-)own the practice where they provide care. Associate GPs generally earn a proportion of the profit or revenue from each visit. For tax purposes, associate GPs are considered firm with their incomes considered profit; therefore, they are not subject to payroll tax [20]. Salaried GPs and GPs on other contracts with a practice are employed by practice-owners and earn all their income through an hourly wage, sessional payment, or annual salary. Contractors working mainly for a single employer are considered employees for tax purposes and are liable for payroll tax [20].

The choice of location by a GP is determined by the utility that is derived from practising in that location. The variables included in the utility functions of previous studies on the observed location choices of doctors include expected earnings, community size, medical resources, the socioeconomic status of the area, and to some extent the leisure amenities of the locations [3, 7, 9, 15].

The aim of this paper is to focus on GPs’ preferences for practice location, specifically differences in job conditions, rather than lifestyle preferences. This is because attributes related to employment are likely more amenable to policy influence. Given that the vast majority of job changes in urban settings do not entail residential mobility,^2^ we restrict the data to mobility within metropolitan locations to enable a focus on job characteristics. The particular focus is how differences in job characteristics between areas of different socioeconomic status affect the location choices of GPs. The analysis focuses on the choice of working in a neighbourhood with low-, medium-, or high-SES, and these form the main choice alternatives.

GPs are assumed to maximise utility when choosing a practice location. We allow for heterogeneity in preferences between practice-owning and salaried GPs. Each location alternative represents a bundle of different job characteristics (i.e. income, workload). The job characteristics included are based on previous utility maximising location choice models [3, 7, 9, 15] and DCEs pertaining to location choice [12, 29, 34]. The specification of the choice models draws on Lancaster’s economic theory of value [18] with preferences modelled using the random utility maximisation framework (RUM) [22]. The flexible RUM framework allows location choice to be a function of a deterministic and stochastic utility component. The model we estimate is for GPs who are already practising, and so GPs face a potential relocation decision in each period.

**Fig. 1:**
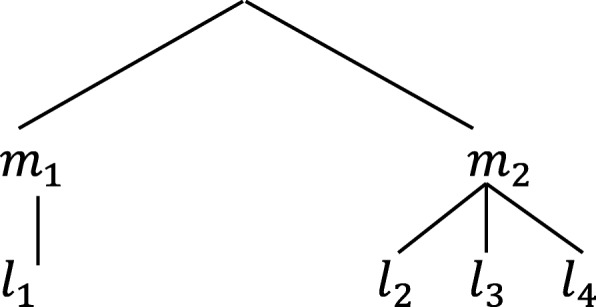
Two-tier nested structure of joint mobility and location choice

Mobility (denoted *m*_*k*_) is the decision to change location (with *k*=1 when the choice is stay and *k*=2 when the choice is to change location). Mobility and location choice (*l*_*1*_,*l*_*2*_*,…l*_*J*_) are related stages of the relocation decision of GPs. Relocation choice therefore naturally lends itself to a nesting structure where the alternatives associated with new locations are more alike than the alternative of staying at the current location. Within each nest, independence of irrelevant alternatives (IIA) is assumed. Figure 1 presents a two-tier nested logit model of mobility and location similar to a model for joint residential mobility and location choice presented by Lee and Waddell [19]. This structure forms the basis of the analysis presented in this paper.

Although the model is tiered, it does not impose a temporal sequence on the decision process. As a result, a decision to relocate can be attributed to a change that causes dissatisfaction with the current location, and therefore, alternative locations are sought (i.e. push factors), or it could be attributable to an alternative becoming exogenously more attractive (i.e. pull factors). The stay branch in this model (where *k* = 1) is a degenerate branch without any twigs.

GP *i* faces the choice between *four* alternative locations (*current location*, *low-SES*, *medium-SES* or *high-SES*). The utility that the GP gains from location *l*_*j*_ is decomposed into a deterministic part, *Y*_*ij*_, and a stochastic part, *ε*_*ij*_. In addition, there is a utility gain (or loss) given that the location choice will require the GP to move or stay; this is captured by *W*_*ik*_. The utility function of the GP is therefore:

1 $$ {U}_{ij}={W}_{ik}+{Y}_{ij}+{\varepsilon}_{ij} $$

*W*_*ik*_ depends on the variables that affect the level of utility loss from moving and thus the choice to move (*k* = 2) or stay (*k* = 1). These factors differ over nests but not over the alternatives within each nest (e.g. if older GPs are less likely to move, then age equally affects all the choices that involve a relocation). *Y*_*ij*_ depends on the characteristics that describe the location alternative *j*, and these characteristics vary over all alternative locations. Unobserved relocation cost is absorbed by the error term in the move branch of the model.

Decomposing utility as described in Eq. 1 means the nested logit probability can be written as a product of two standard logit probabilities. With the probability of choosing location *j* being expressed as the product of the probability that relocation is chosen (${P}_{im_2}$) and the probability that alternative *j* is chosen conditional on relocation (${P}_{ij\mid {m}_2}$):

2 $$ {P}_{i1}={P}_{i{m}_1} $$

3 $$ {P}_{ij}={P}_{ij\mid {m}_2}{P}_{im_2}\ \mathrm{for}\ j=2,\dots, J $$

where:

- ${P}_{im_1}$ = marginal probability of GP *i* staying in their current location
- ${P}_{im_2}$ = marginal probability of GP *i* relocating
- ${P}_{ij\mid {m}_2}$ = conditional probability of location *j* given the choice to relocate

Location choice is determined using the marginal probability (choice to relocate) and the conditional probability (location choice within the move nest). In the model presented in this paper, a logit specification is chosen for these probabilities. They can be written as:

4 $$ {P}_{im_k}=\frac{{\mathrm{e}}^{W_{ik}+{\lambda}_k{L}_{ik}}}{\sum_{j=1}^K{\mathrm{e}}^{W_{ij}+{\lambda}_j{L}_{ij}}} $$

5 $$ {P}_{ij\mid {m}_k}=\frac{{\mathrm{e}}^{Y_{ij/{\lambda}_k}}}{\sum_{r\epsilon {m}_k}{\mathrm{e}}^{Y_{ir/{\lambda}_k}}} $$

with:

6 $$ {L}_{ik}=\ln \sum \limits_{j\epsilon {m}_k}{\mathrm{e}}^{Y_{ij/{\lambda}_k}} $$

*L*_*ik*_ is the inclusive value of the choice to move (where *k* = 2) or stay (where *k* = 1); it links the conditional and marginal probabilities. *λ*_*k*_*L*_*ik*_ captures the utility to GP *i* of the alternatives in the nest *m*_*k*_, with the coefficient of the inclusive value, *λ*_*k*_, being the log-sum coefficient. In order to get consistent and efficient estimates, simultaneous maximum likelihood estimation is used (see [31]).

Both nested and mixed nested logit estimation were considered for the relocation model. The Bayesian Information Criterion (BIC) and log-likelihood ratios favour the nested logit structure; therefore, a closed-form nested logit model is estimated. Using the nested logit model, the average marginal effects of the variables are computed by averaging the individual marginal effects over all observations.

## Data

Data from the Medicine in Australia: Balancing Employment and Life (MABEL) panel survey are used [17]. This is a panel survey of around 10 000 doctors each year, including around 4000 GPs. We observe data on the locations of responding GPs from 2008 to 2011. The MABEL sample has been shown to be broadly representative of age, gender, hours worked, and location [17]. Registrars, locums, GPs working in other non-traditional settings and GPs seeing fewer than ten patients per week or working less than 7 h per week are excluded to generate a sample of actively practising GPs. This results in a pooled sample of 3426 GP observations from metropolitan areas where a GP move can be observed; given the use of mobility as the dependent variable, all GPs included in the model need to be observed more than once across the 4 years. Metropolitan areas were defined using the Australian Standard Geographic Classification (ASGC) of major cities.

The MABEL survey questionnaire includes questions on the postcode of work. Relocation is defined as changing the postcode of main practice of work between two-time periods. A GP that changes location within the same postcode is not captured as a relocation, and those quitting general practice are excluded from the sample. It is not possible to identify when an entire GP practice relocates; therefore, such relocations are counted in the same manner as a change of job. Limiting the sample to metropolitan areas results in mobility likely to be just a change of job; 84% of the mobility in the sample involves only practice relocation (with residential postcode location staying the same). Given the sample is restricted to metropolitan areas, we assume these residential relocations do not result in large lifestyle changes.^3^

SES is based on the Australian Bureau of Statistics (ABS) Index of Relative Socioeconomic Disadvantage (IRSD) score [1]. The IRSD captures the economic and social disadvantage (i.e. low income, low education, high unemployment, lone parents) of households within an area. Socioeconomic status is grouped into three categories which represent the bottom 30% of IRSD scores (deemed low SES), the middle 40% (deemed middle SES), and the top 30% (deemed high SES).

Descriptive statistics and brief definitions are presented in Table 1. The GP’s personal characteristics included are age, gender, marital status, having dependent children at home, and medical degree from an Australian versus international medical school.

**Table 1:** Descriptive statistics (*N* = 3426 GP observations)

Variable	Mean (standard deviation)	Definition
Age	52.51 (10.15)	Self-reported year of birth.
Female = 1	0.47 (0.50)	Binary gender variable. MABEL survey.
Living with partner = 1	0.88 (0.33)	Binary variable. Response to “Are you currently living with a partner or spouse?” MABEL survey.
Number of Dependent children	0.62 (0.33)	Count variable. Derived from the reported number of dependent children. MABEL survey.
Australian qualified = 1 MD	0.81 (0.40)	Binary variable. Responded they completed their medical degree in Australia. MABEL survey.
Consultation length (min)	16.42 (6.80)	Number of minutes the average consultation lasts. MABEL survey
Volume (patients per week) adjusted for number of days worked	110.03 (60.14)	Response to “In your most recent USUAL week at work, for around HOW MANY patients did you provide care?” MABEL survey.
Patient complexity	2.77 (1.02)	Rank of strongly disagree to strongly agree (5-point scale) with the statement “the majority of my patients have complex health and social problems”.
On-call = 1	0.33 (0.47)	Binary variable. Capturing doing after-hours and on-call.
Gross annual earnings (AU$)	184 976 (120592)	Self-reported gross earnings. MABEL survey.
GPs per 10 000 persons	17.44 (12.66)	The number of GPs in the postal code of the GP’s main practice location as reported by AMPCo [2] divided by the population in that postal code as reported by the ABS.
Practice-owner = 1	0.46 (0.50)	Binary variable. Derived from GPs self-reported relationship with practice (Principal/partner; associate; salaried employee; contracted employee)

Given the heterogeneity in hours that most GPs experience week on week, we focus on the number of days worked per week (with each day assumed to be 7 h of work). The data show that 23% of the pooled sample changed the number of days worked per week over the survey; however, only 3% of GPs changed the number of days worked per week and their location simultaneously. Since DCEs have demonstrated that after-hours or on-call work is undesirable to GPs [34], we take this into account by capturing the proportion of GPs that report doing any after-hours (public holidays, weekends, and weekdays outside of 8 am to 6 pm) or on-call work.

The analysis allows preferences to differ between practice-owning and employee GPs. The sample data suggests that GPs do not commonly change practice mode. Only 4.4% changed practice mode over the 4 years observed, with 1.9% (approximately 65 GPs) becoming a practice owner and 2.5% (approximately 85 GPs) of practice-owning GPs becoming employees.

Evidence suggests that workload varies systematically across different locations. GPs practising in low-SES areas in Australia face higher demand levels, have less time, see patients with more co-morbidities, and experience greater levels of stress than those practising in high-SES areas [33]. Therefore, workload variables such as consultation length, patient volume and patient complexity factor into location choice. Van Ryn and Burke [32] demonstrate that physicians view patients with low or middle socioeconomic status more negatively than patients with higher SES. Further, Britt et al. [4] demonstrate that patients with higher SES tend to receive longer consultations than those with lower SES. Consultation length could, therefore, also be associated with the SES of the location. In this paper, consultation length is measured through GPs’ responses to the question “how long does the average consultation last?” Patient volume is related to demand and demand to SES. However, patient volume is also likely to be associated with consultation length; higher volume practices will certainly have shorter consultations unless they compensate with longer hours. To avoid double counting consultation length and patient volume, and to allow for the number of patients GPs treat to expand at the margins, patient volume is measured as patients per week (rather than patients per hour). This variable is then adjusted for the number of days the GP works per week to calculate a full-time equivalent rate. Patient complexity is the GPs’ response to how much they agree/disagree on a 5-point scale (with an opt-out “not applicable” available) with the statement that “the majority of my patients have complex health and social problems”.

All GPs in MABEL were asked to provide their gross earnings. Using annual earnings should enable comparisons between practice-owning and employee GPs. This annual earnings approach (rather than hourly wage) is often used in DCEs (see for example [34]).

Finally, GP density is included to capture local competition. GP density is defined as the number of GPs in each postcode per 10 000 persons in the postcode.

If a GP stays at their current location in year *t*, the stay branch is populated with data on the characteristics of their actual job in year *t*. Counterfactual data need to be determined for the “move” nest. Data for expected earnings and patient volume are adjusted based on the prior decision of number of days worked per week; therefore, a GP currently working 3 days per week faces a different set of counterfactuals than a GP currently working 5 days. Variation in GP earnings can be observed across states and SES in Australia [6]. Counterfactual data are based on mean state (of which there are eight in Australia) level data for each neighbourhood type (low, medium, and high socioeconomic status) for each year used in the model. That is, the counterfactual choice set a GP in one state faces is different from the choice set in a different state and they are dynamic; that is, they change relative to the mean observed data for each period.

Using the panel nature of the data, the actual characteristics at the observed location choice (i.e. the observed location at time *t*) of the GP for time *t* are used. For GPs who move, data on the observed location choice (i.e. the new location at time *t*) and data from their previous observed location, their location choice at time *t* − 1, are used alongside the counterfactual data (the mean characteristics by state and year) for the two remaining location choice options. Therefore, if a GP stays at their current location, the stay branch is populated with data of that location from the current time period while for the three choices in the move branch, mean values by state for the current year are used.

## Results

Between 2008 and 2011, approximately 6% (213) of the observed metropolitan GP sample made a decision to relocate. This suggests that transaction costs related to relocation or other factors such as status quo bias play an important role in relocation choices. In addition, it appears that metropolitan GP mobility is slightly lower than that observed for the entire GP population [21, 23]. Table 2 presents the relocation choices of GPs in the sample. The vast majority (approximately 94%) of GPs chose to remain at their current location. This is roughly equal for all GPs regardless of the SES of their initial location. Only 9% of GPs who made a choice to move relocated to a neighbourhood with low-SES, which poses methodological challenges for the policy simulations. Fifty-five percent of movers relocated to a neighbourhood with high-SES. Most of the relocations were towards a neighbourhood with the same SES as the current location, only 21% of moves were towards a neighbourhood with lower SES, and 28% were towards a neighbourhood with a higher SES.

**Table 2:** Transition table: relocation choice

		Stay (t)	Move to low SES (*t*)	Move to middle SES (*t*)	Move to high SES (*t*)	Total
Low SES (*t* − 1)	Number	401	4	10	13	428
	% total	93.7%	0.9%	2.3%	3.1%	
	% movers		15%	37%	48%	27
Middle SES (*t* − 1)	Number	1092	6	38	36	1172
	% total	93.2%	0.5%	3.2%	3.1%	
	% movers		7%	48%	45%	80
High SES (*t* − 1)	Number	1720	9	29	68	1826
	% total	94.2%	0.5%	1.6%	3.7%	
	% movers		9%	27%	64%	106
Total	Number	3213	19	77	117	3426
	% total	93.8%	0.6%	2.2%	3.4%	
	% movers		9%	36%	55%	213

The results for the nested logit models on relocation decisions are presented in Table 3. The estimated log sums for the move nest are small and statistically significant (approximately 0.24) in all the model specifications. This provides strong support for the nested logit model specification and suggests that substitution within the move nests is greater than substitution between the move and stay nests.

**Table 3:** Nested logit model of relocation choice

Variable	Model 1	Model 2	Model 3
Mobility (base case: stay)			
Practice-owner	− 1.425*** (0.178)	− 3.523** (1.406)	− 1.70*** (0.237)
Age	− 0.038*** (0.004)	− 0.037*** (0.005)	− 0.037*** (0.004)
Age*Owner		0.023 (0.021)	
Female	0.169 (0.145)	0.063 (0.165)	0.038 (0.163)
Female*Owner		0.709** (0.352)	0.644* (0.344)
Living with partner	0.066 (0.201)	0.065 (0.654)	0.082 (0.200)
Spouse*Owner		0.477 (0.654)	
Dependent children	− 0.184 (0.151)	− 0.198 (0.173)	− 0.181 (0.150)
Children*Owner		0.291 (0.392)	
Australian MD	− 0.228 (0.161)	− 0.150 (0.183)	− 0.181 (0.161)
Australian MD*Owner		− 0.091 (0.413)	
Location attributes			
Consult length (min)	0.024** (0.010)	0.018 (0.012)	0.018** (0.009)
Consult length*Owner		− 0.003 (0.017)	
Volume (patients per week)	0.003** (0.001)	0.002 (0.001)	0.002 (0.001)
Volume*Owner		− 0.001 (0.002)	
Patient complexity	− 0.119** (0.048)	− 0.180*** (0.053)	− 0.186*** (0.052)
Patient complexity*Owner		0.306*** (0.099)	0.316*** (0.094)
On-call	− 0.053 (0.104)	0.045 (0.113)	0.056 (0.112)
On-call*Owner		− 0.352* (0.208)	− 0.362* (0.210)
Earnings (log)	0.432*** (0.052)	0.433*** (0.056)	0.425*** (0.053)
Earnings*Owner		− 0.097 (0.117)	
GPs per 10 000 persons	0.011*** (0.004)	0.012*** (0.004)	0.010*** (0.004)
GPs/10000*Owner		− 0.007 (0.008)	
Move nest (log sum)	0.272*** (0.044)	0.239*** (0.041)	0.242*** (0.041)
Observations	3 426	3 426	3 426
BIC	1877.599	1962.808	1893.074
AIC	1 772.243	1 774.672	1 765.141
*x*^2^ *p*-value	0.000	0.000	0.000
Log-likelihood	− 872.122	− 862.336	− 865.571

Variable coefficients with standard errors in parentheses

*Significant at the 10% level; **significant at the 5% level; ***significant at the 1% level

Model 1 in Table 3 presents a model including practice ownership as a personal characteristic but does not include interactions between ownership and other variables. Model 2 presents a model with interactions between practice ownership and all the included variables; this model allows for an assessment of the relationship between mobility and practice ownership while considering that practice owners have different characteristics and some of these may also affect preferences. Results suggest that female practice owners were the more mobile than female employees and that practice owners were more attracted to areas with higher patient complexity. Model 3 presents a more parsimonious model with interaction terms for the variables that had a statistically significant interaction term in model 2. Model 3 is deemed the model that best fits the data (lowest Akaike Information Criterion [AIC] and likelihood ratio tests). However, given the degree of subjectivity involved in this choice, Table 3 presents coefficients for all models. Results are reasonably stable across the three model specifications.

Overall, the estimated parameters in Table 3 suggest that younger and employee GPs are more likely to relocate than older and self-employed GPs. The job characteristics across each alternative show that expected earnings are positively associated with utility, but non-pecuniary attributes are also important. GPs prefer less complex patients, longer consultation lengths, and perhaps surprisingly, work in areas where there are more GPs per capita.

## Discussion

The marginal effect sizes (Table 4) provide an indication of the importance of each variable in the choice to relocate. The average marginal effects demonstrate that a 1% increase in the expected earnings of GPs in a low-SES neighbourhood would increase the probability of mobility by 2 percentage points. Being 1-year older results in a decreased probability of relocation by 0.31 percentage points and being a practice-owner is associated with a reduced probability of mobility of 1.7 percentage points.

**Table 4:** Average marginal effects based on the nested relocation choice model

Variable	AME
Mobility (base case: stay)	
Practice owner	− 1.731***
Age	− 0.310***
Female	0.171
Female*Owner	1.913
Living with partner	0.128
Dependent children	− 0.444
Australian MD	− 0.25
Location attributes	
Consult length (min)	0.093
Volume (patients per week)	0.008
Patient complexity	− 0.941***
Patient complexity*Owner	0.487
On-call	0.233
On-call*Owner	− 0.195
Earnings (log)	2.161***
GPs per 10 000 persons	0.005*

AME presented in percentage points. Estimated by bootstrapping 500 repetitions

*Significant at the 10% level; **significant at the 5% level; ***significant at the 1% level

Table 5 summarises the actual and predicted probabilities of each of the branches in the model using the sample of 3426 GP observations. Most GPs (93.8%) stay in their current location. The model performed well in predicting the probability of a GP choosing to relocate, with approximately 6% of the sample observed and predicted to relocate between 2008 and 2011. Of those who relocate, moving into a middle- or high-SES area is more likely than relocating to a low-SES area; only 0.6% of the observed sample and 1.9% of the simulated sample relocated into neighbourhoods with low socioeconomic status.

**Table 5:** Actual versus predicted choices

Location choice	Actual	Predicted
Stay	93.8	93.6
Move to low SES	0.6	1.9
Move to middle SES	2.2	2.2
Move to high SES	3.4	2.3

Percentage of total

Establishing the effectiveness of relocation incentives on GPs already in the labour market is important when considering the overall effectiveness of redistribution policies. Therefore, the parsimonious model is used to run a policy simulation of an income-loading redistribution policy on GPs’ location choice, including the choice to relocate or remain at the current practice and, if relocation is chosen, the SES of that neighbourhood. The simulation presented in Table 6 predicts that a policy increasing the earnings of all GPs in low-SES neighbourhoods by 10% results in 0.79 percentage points more GPs (approximately 123 GPs) being retained or relocating to low-SES neighbourhoods than would have in the absence of the policy. Table 7 shows that the income loading policy has higher impact on employee GPs than practice-owning GPs. A 10% increase in the earnings of all metropolitan GPs practising in low-SES neighbourhoods would cost approximately AU $50 million per year.

**Table 6:** Predicted probabilities before and after the 10% increase in earnings to GPs located in areas with low SES (change attributed to policy simulation in parentheses)

Location choice before reform	Location choice after reform		
	Low SES	Middle SES	High SES
Low SES	95.9% (+ 0.30)	2.1% (− 0.15)	2.0% (− 0.14)
Middle SES	2.2% (+ 0.25)	95.5% (− 0.16)	2.3% (− 0.07)
High SES	2.1% (+ 0.24)	2.1% (− 0.09)	95.8% (− 0.15)

**Table 7:** Practice-owning GPs compared to employee GPs: predicted probabilities before and after the 10% increase in earnings to GPs located in areas with low SES (change attributed to policy simulation in parentheses)

GP type	(Re)locating in low SES from		
	Low SES	Middle SES	High SES
Practice-owning GPs	98.1% (+ 0.15)	1.1% (+ 0.12)	1.2% (+ 0.14)
Employee GPs	93.8% (+ 0.42)	3.2% (+ 0.36)	2.8% (+ 0.32)

However, a limitation of these results is that the model provides a good estimate of the overall predicted mobility of GPs, but not of mobility into different types of neighbourhood. This is likely due to the small proportion of moves. In order to build a model able to capture this, there would need to observe more relocations. Although the sample of 3426 GPs is the largest sample of observed location choice of doctors in the literature (Hurley [15] and Bolduc et al. [3] both had samples of about 900 newly entering doctors), the proportion relocating into low-SES neighbourhood is too low to simulate this choice with high precision.

The study demonstrates the heterogeneity in mobility by GP characteristics such as age, gender and practice ownership. Although improvements to the geographic targeting of recruitment and retention incentives [16] have been recommended, the results of this study suggest that targeting incentives to GPs with certain characteristics that are more amenable to relocation (i.e. younger GPs and employee GPs) may result in better uptake of incentives.

## Conclusion

The results highlight that financial considerations are part of a larger number of factors influencing location choice. For instance, practice ownership played almost as important a role in mobility as earnings. This research has provided valuable evidence on the potential role of financial incentives in influencing location choices. Previous policy simulations suggest financial incentives aimed at locating new doctors in specific areas could be an effective policy lever [3, 15]. This paper finds that established GPs are not very mobile, even when a financial incentive is offered. The simulations presented in this paper, although limited, suggest that financial incentives, which are being used widely, may have limited effectiveness in inducing GPs to relocate once they have made an initial location choice. This seems to be reflected by the current geographic distribution of physicians, even with financial incentive programmes being adopted by many countries.

## Abbreviations

ABS: Australian Bureau of Statistics
AIC: Akaike Information Criterion
ASGC: Australian Standard Geographic Classification
BIC: Bayesian Information Criterion
DCEs: Discrete choice experiments
GPs: General Practitioners
IIA: Independence of irrelevant alternatives
IRSD: Index of Relative Socioeconomic Disadvantage
MABLE: Medicine in Australia: Balancing Employment and Life
MADA: Medical and Dental Accounting
SES: Socioeconomic status
WHO: World Health Organization

## Footnotes

1 Estimates derived from the Medicine in Australia Balancing Employment and Life (MABEL) Wave 1 (2008) data on GPs.

2 The MABEL data show that in metropolitan areas only 16% of the jobs mobility was accompanied by a residential move.

3 Excluding the 16% who had a residential and practice relocation in the same year does not change the concluding results.
